# Dr. Frederic Clift

**Published:** 1913-05

**Authors:** 


Dr. Frederic Clift, Keokuk, 1891, died at Kaysville, Utah, March 28, of pneumonia, aged 65. He was Secretary of the Davis Co. Medical Association and Editor of the *Utah Medical Journal,* the last number of which noted the absence of certain editorial and other matter due to his sickness.

